# SNS-032 inhibits mTORC1/mTORC2 activity in acute myeloid leukemia cells and has synergistic activity with perifosine against Akt

**DOI:** 10.1186/1756-8722-6-18

**Published:** 2013-02-18

**Authors:** Haitao Meng, Yingming Jin, Hui Liu, Liangshun You, Chunmei Yang, Xue Yang, Wenbin Qian

**Affiliations:** 1 Institute of Hematology, The First Affiliated Hospital, College of Medicine, Zhejiang University, 79# Qingchun Road, Hangzhou, 310003, P. R. China

**Keywords:** SNS-032, mTORC1, mTORC2, Cyclin-dependent kinases, Perifosine, Akt, Acute myeloid leukemia

## Abstract

**Background:**

Acute myeloid leukemia (AML) is a heterogeneous disorder with aberrant regulation of a variety of signal pathways. Therefore, simultaneous targeting of two or even more deregulated signal transduction pathways is needed to overcome drug resistance. Previously, it was reported that SNS-032, a selective cyclin-dependent kinase inhibitor, is an effective agent for treatment of AML; however, the molecular mechanisms of SNS-032-induced cell death of AML cells are not yet fully understood. The aim of the study was to characterize the effects in vitro of SNS-032, used alone and in combination with an Akt inhibitor perifosine, against AML cells and to identify the mechanism involved.

**Results:**

SNS-032 significantly induced cytotoxicity in human AML cell lines and blasts from patients with newly diagnosed or relapsed AML. However, Kasumi-1 cells and some of leukemic samples (14.9%) from AML patients were resistant to SNS-032-mediated cell death. Western blot analysis showed that SNS-032 strongly inhibited the phosphorylation of mammalian target of rapamycin (mTOR) on Ser 2448 and Ser2481, and that removal of SNS-032 resulted in partial recovery of cell death and reactivation of phosphorylation of mTOR. Moreover, exogenous insulin-like growth factor-1 (IGF-1) did not reverse SNS-032-induced cell growth inhibition and downregualtion of phosphor-mTOR at Ser2448 and Ser2481 although slight suppression of IGF-1R expression was triggered by the agent. Furthermore, SNS-032 at a lower concentration (60–80 nM) enhanced AML cell cytotoxicity induced by perifosine, an Akt inhibitor. Importantly, SNS-032 treatment reduced colony formation ability of AML cells, which was significantly increased when two agents were combined. This combination therapy led to almost complete inhibition of Akt activity.

**Conclusion:**

We conclude that SNS-032 might directly target mammalian target of rapamycin complex 1 (mTORC1)/mTORC2. Our results further provide a rationale for combining SNS-032 with perifosine for the treatment of AML.

## Introduction

Acute myeloid leukemia (AML) is an aggressive malignancy that can be characterized by rapid growth of a clonal population of neoplastic cells that accumulate in the bone marrow as a result of a blockage in hematopoiesis. In spite of many efforts in the past decades, the outcome for the patients remains poor. AML is predominantly a disease of the elderly. Long-term survival is achieved by approximately 40%-45% of younger patient with AML but less than 10% of patients aged >60 years [1,2]. Thus new therapeutic approaches should be explored in the hope of improving outcomes.

AML is a very heterogeneous disease with the constitutive activation of signal transduction pathways that enhances the survival and proliferation of the leukemic cells [3]. With marked improvements in our understanding of the molecular events occurring during the development of AML, the number of potential targets for therapy has grown rapidly [4]. For example, numerous small molecular inhibitors as monotherapy or in combination with chemotherapy, including Fms-like tyrosine kinase 3 (FLT-3) inhibitor (sorafenib), farnesyl-transferase inhibitor (tipifarnib), histone deacetylase inhibitor (vorinostat), as well as DNA methyltransferase inhibitors (decitabine, azacitidine), are already on clinical trial for AML [4,5].

The cyclin-dependent kinases (CDKs), a family of serine/threonine kinases, regulate cell cycle events and some members are associated with transcription control. CDK activity is often perturbed in cancer cells but not in human normal cells. This tumor-specific deregulation makes the CDKs being a major target for therapy [6,7]. SNS-032 is a potent and selective inhibitor of CDK2, –7, and −9 [7]. It has been reported that the antitumor effects of SNS-032 are observed in a variety of solid tumors and hematopoietic malignancies such as chronic lymphocytic leukemia (CLL) [8], mantle cell lymphoma (MCL) [9], and chronic myeloid leukemia (CML) [10]. These studies have led to the phase I evaluation of SNS-032 as a potential therapy for CLL and multiple myeloma [11]. More recently, Walsby E, *et al.*[12] reported that SNS-032 effectively inhibited proliferation of NB4, HL-60 cells and fresh AML samples by inducing a marked dephosphorylation of Ser2 and Ser5 of RNA polymerase (RNA Pol) II carboxy terminal domain (CTD) and inhibiting the expression of CDK-2, and −9. Furthermore, cotreatment with SNS-032 and cytarabine (Ara-c) resulted in remarkable synergy that was associated with reduced expression of the antiapoptotic genes xIAP, Bcl-2, and Mcl-1. Although it has been demonstrated that SNS-032 is capable of inducing cell death in CLL and MCL cells via inhibition of CDKs that regulate the initiation and elongation of transcription and decrease of the levels of short-lived proteins such as xIAP, Bcl-2, Mcl-1, and cyclin D1 [8,9], the molecular mechanisms underlying the response of the AML cells to SNS-032 are not fully understood. In this study, we addressed the molecular mechanisms of the antileukemia action of SNS-032. Our results show that SNS-032 significantly inhibits cell proliferation and induces apoptosis in AML cells. However, some of leukemic cells are resistant to the drug-induced cell death. Furthermore, we show, for the first time, that SNS-032 suppresses the levels of mTOR expression and phosphor-mTOR on Ser2448 and Ser2481. Additionally, treatment of human AML cells with SNS-032 in combination with Akt inhibitor perifosine causes enhanced cell death. This synergistic cytotoxic effect most likely results from elimination of Akt activation. The findings of the present study provide a rationale for combining SNS-032 with perifosine for the treatment of AML.

## Results

### SNS-032-mediated leukemia cell-killing effect

**Figure 1:**
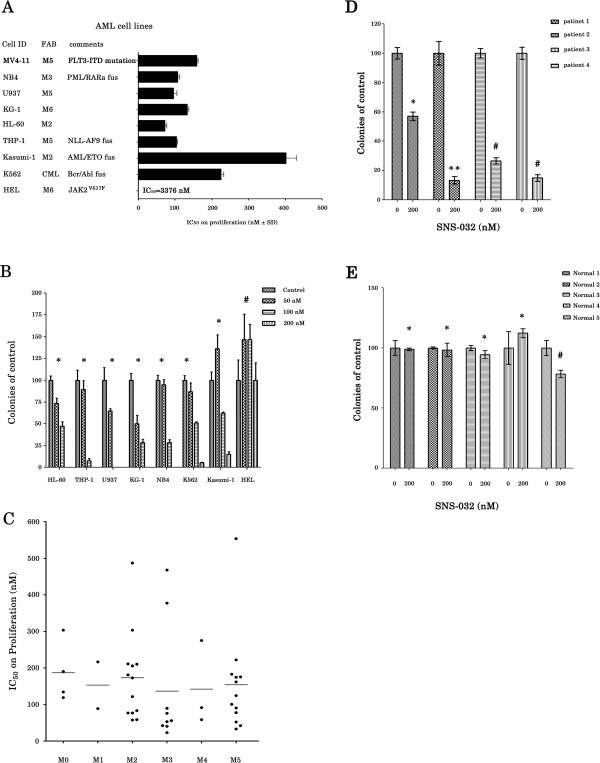
**SNS-032 potently inhibits cell growth in AML cell lines and primary AML blasts. **(**A**) IC_50 _of cell lines tested in 24 h by an MTT assay. Data show mean ± s.d. from three independent experiments, each performed in triplicates. The comments of cell lines are shown. (**B**) After treatment with 50, 100, or even 200 nM SNS-032 for 7 days, the ability of colony formation of all tested cell lines were evaluated in methylcellulose cultures. All data represent an independent experiment from three repeated tests with similar results. Each point is the mean of triplicates; bar, ± s.d. * represent *P* < 0.0001; #*P* = 0.31. (**C**) IC_50_ of primary AML cells from the patients with different French-American-British (FAB) classification subtypes was examined in 48 h by colorimetric assay. (**D**) Primary leukemic cells from 4 patients with newly diagnostic AML were treated with SNS-032 (200 nM) and then cultured in methylcellulose medium containing rhSCF, rhGM-CSF and rhIL-3 for 7 days. Colonies were counted under a microscope. * *P* = 0.0009; ** *P* = 0.0005; # *P* < 0.0001. (**E**) Human normal mononuclear cells obtained from bone marrow of 5 healthy volunteers were incubated with or without SNS-032, and then cultured in methylcellulose medium for 12 days to detect colony formation. * represent *P* > 0.05; # *P* = 0.0052.

It has been shown that AML and CML cells are sensitive to SNS-032 [10,12]. We first examined the effect of SNS-032 on the viability of cultured AML cell lines. As shown in Figure 1A, the doses that inhibited 50% proliferation (IC_50_) at 24 h on cell proliferation in a panel of 7 AML cell lines ranged from 71.7-402 nM, with the panel including subtypes M2, M3, M5, and M6 according to the French-American-British (FAB) classification. The IC_50_ in CML K562 cells was 224.3 nM. HEL cells, however, were found to be resistant with IC_50_ > 3000 nM. Consistent with these results, colony formation assay showed that a significant reduction in clonogenic ability at 50 and 100 nM and a complete cessation of colony formation at 200 nM in HL-60, THP-1, U937, KG-1, and NB4 cells, but not in Kasumi-1 and K562 cells. HEL cells were resistant to SNS-032 in respect to inhibiting colony forming (Figure 1B). We next evaluated the effects of SNS-032 on the cellular proliferation of primary leukemic cells. The characteristics of 47 patients are detailed in Table 1. The majority (85.1%) of primary AML samples was very sensitive to the drug, with mean IC_50_ values for the different FAB types ranging between 136.2 nM and 186.7 nM (Figure 1C). There was no significant difference between the response to SNS-032 and the characteristics of AML patients (Table 1). However, a small fraction (14.9%) of the specimens was relatively resistant to SNS-032-mediated cell death (IC_50_ >300 nM). Also, a significant decrease in the number of colony formation was observed in the primary blasts obtained from 4 patients with newly diagnostic AML (Figure 1D), but not in the bone marrow cells from healthy volunteers (Figure 1E).

**Table 1:** Patients characteristics

**Characteristic**	**Number (%)**	**Mean IC**_**50 **_**(nM)**	***P *value**
**Sex**			0.204*
Male	29 (62)	175.0 ± 127.0	
Female	18 (38)	138.7 ± 126.1	
**FAB classification**			0.404
M0	4 (9)	186.7 ± 83.7	
M1	2 (4)	152.7 ± 89.9	
M2	14 (30)	156.4 ± 124.6	
M3	9 (19)	136.2 ± 165.2	
M4	3 (6)	141.7 ± 116.5	
M5	13 (28)	153.1 ± 134.5	
M6	2 (4)	289.9 ± 111.8	
M7	0 (0)		
**Type of AML**			
De novo	44 (94)	154.4 ± 115.6	
Relapsed	2 (4)	322.7 ± 326.7	
MDS	1 (2)	134.1	
**Cytogenetic class**			0.092
Favorable	8 (17)	114.7 ± 121.3	
Intermediate	31 (66)	153.5 ± 121.9	
Unfavorable	5 (11)	168.6 ± 134.5	
Unknown	3 (6)	143.2 ± 65.7	
**Flt3 ITD status**			0.092
Wild-type	23 (49)	133.1 ± 87.6	
Mutated	4 (8)	89.6 ± 63.4	
Unknown	20 (43)	207.6 ± 157.9	
**NPM1 status**			0.135
Wild-type	23 (49)	124.7 ± 81.7	
Mutated	4 (8)	137.8 ±115.0	
Unknown	20 (43)	207.6 ± 158.0	

*For comparison, chi-square test was used.

### SNS-032 induced apoptosis and inhibited not only phosphorylation of RNA Pol II but also phosphorylation of mTOR and its downstream targets

**Figure 2:**
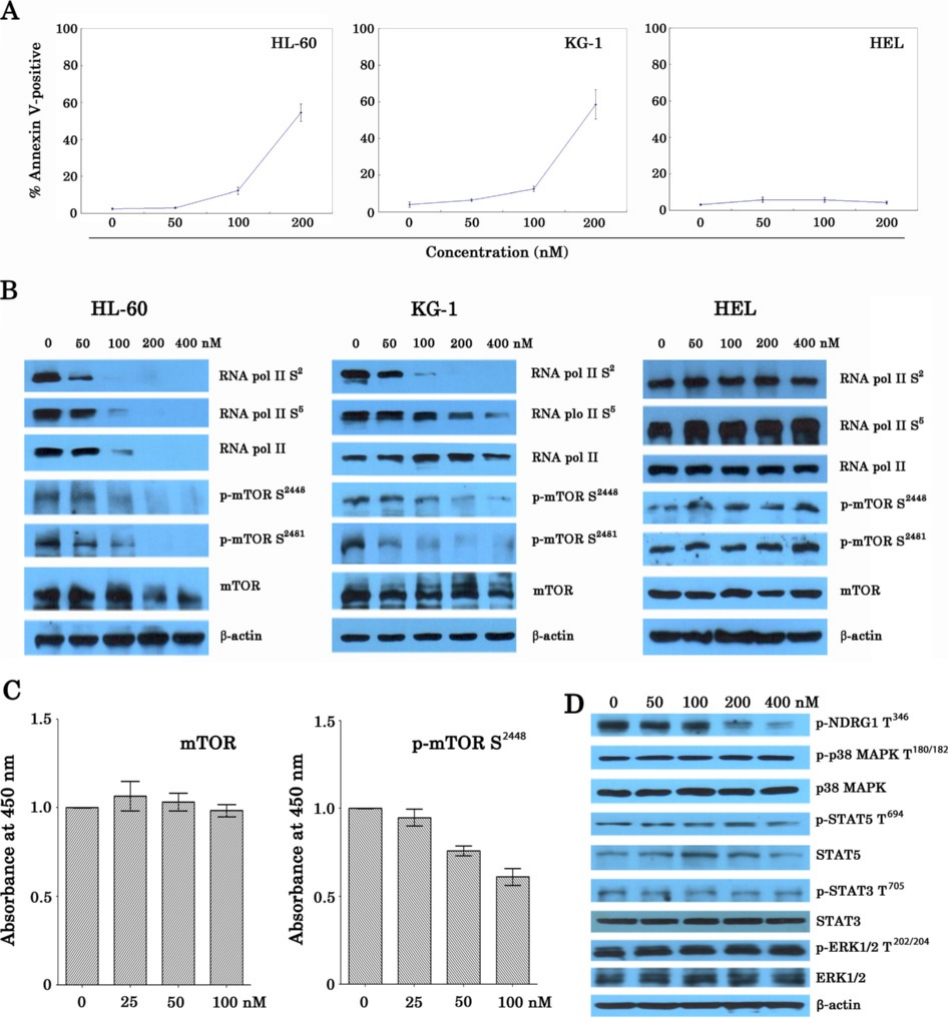
**Induction of apoptosis and inhibition of phosphorylation of RNA Pol II and phosphor-mTOR by SNS-032. **(**A**) HL-60, KG-1 and HEL cells were treated with SNS-032 for 24 h, and apoptosis was assessed by flow cytometry after staining of the cells with annexin V-FITC and propidium iodide. (**B**) Three AML cell lines were treated with SNS-032 at the indicated doses for 6 h. PBS was used as a negative control. The total cell lysates (50 μg of protein per lane) were analyzed by Western blotting with antibodies against RNA pol II, RNA pol II carboxy terminal domain phosphor-serine 2 and phosphor-serine 5, mTOR, phosphorylated form of mTOR on Ser2448 and Ser2481. Anti-β-actin was used as a loading control. (**C**) HL-60 cells were treated SNS-032 at the indicated doses for 6 h. The endogenous levels of mTOR protein phosphorylated at Ser2448 and were detected using solid phase sandwich enzyme-linked immunosorbent assay. (**D**) HL-60 cells were treated with SNS-032 (50–400 nM for 6 h). whole-cell lysates were subjected to Western blotting to assess phosphorylation and protein expression of NDRG1, p38MAPK, STAT3/5, and ERK1/2.

Previous studies showed that induction of apoptosis is a key action for SNS-032-induced cell death in AML and CML [10,12]. We therefore evaluated the effect of SNS-032 on apoptosis of AML cell lines. Cells were treated with increasing doses of the drug for 24 h, and then apoptotic cells were determined by annexin V-FITC. The 50% effective concentration (EC_50_) of KG-1 and HL-60 cell lines was 192.2 and 194.8 nM, respectively. In contrast, HEL cells were resistant to SNS-032-induced apoptosis. There was little cell death at 24 h after SNS-032 treatment, even at concentration of 200 nM (Figure 2A). To examine the cell cycle effects, HL-60 cells were cultured with SNS-032 or Rapamycin, respectively, and cell-cycle analysis was performed. The cells exposed to SNS-032 showed accumulations of cells in G1 phase (data not shown), consistent with prior reports [12] that showing that SNS-032 induces a cell-cycle arrest. The increased percentages of cells in the G1 phases were also observed in HL-60 cells treated with Rapamycin. Next, we set out to unravel the molecular mechanism of action of SNS-032. On western blot analysis, we observed that SNS-032 dose-dependently decreased phosphorylation of RNA pol II at Ser2 and Ser5 in KG-1 and HL-60 cells following 6 h of incubation (Figure 2B). These are consistent with the previous report [12]. Interestingly, we found that SNS-032 strongly inhibited phosphorylation of mTOR on Ser2448, a marker for mTORC1 activity [13], as well as phosphorylation of mTOR protein on Ser2481, a marker for the presence of mTORC2 complexes [13,14]. The activity of mTORC1 and mTORC2 in HL-60 and KG-1 cells was completely inhibited by the treatment with 200 and 400 nM SNS-032 accompanied by slight degradation of protein expression of mTOR (Figure 2B). The downregulation of endogenous levels of mTOR protein phosphorylated at Ser2448 was also confirmed in the treated HL-60 cells using ELISA assays (Figure 2C). To test the effect of SNS-032 on unrelated signaling pathways, immunoblotting analysis was performed (Figure 2D). The addition of the drug did not suppress extracellular signal-regulated kinase (ERK) Thr202/Tyr204 phosphorylation, p38 mitogen-activated protein kinase (MAPK) Thr180/Tyr182 phosphorylation in HL-60 cells, and also did not decrease signal transducer and activator of transcription 5 (STAT5) Tyr694 phosphorylation and STAT3 Tyr705 phosphorylation. These data emphasize the specificity of SNS-032 against mTOR activity.

**Figure 3:**
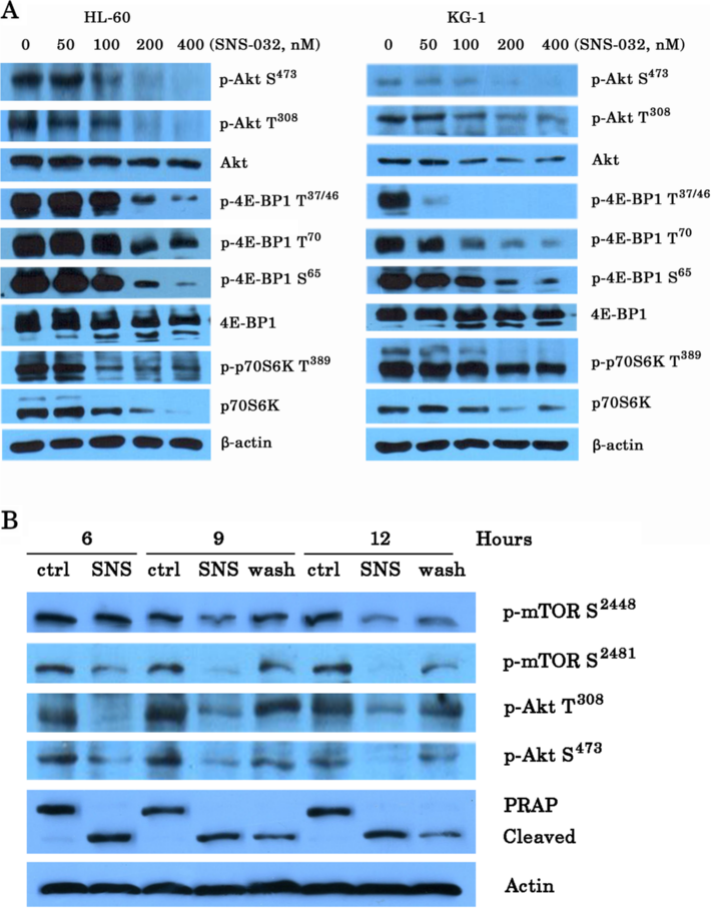
**SNS-032 strongly inhibits expression and phosphorylation of downstream targets of mTOR, and removal of SNS-032 correlates with the recovery from inhibition of phosphor-mTOR and cell death. **(**A**) Exponentially growing cells exposed to the indicated doses SNS-032 for 6 h and then cell lysates were prepared, and 50 μg of total cell extracts was separated in 6-12% SDS-PAGE. Protein bands were detected with antibodies to phosphor-Akt (Ser437/Thr308), Akt, phosphor-4E-BP1 (Ser65/Thr37/46/Thr70), 4E-BP1, phosphor-p70S6K (Thr389), p70S6K, and actin. This experiment was repeated once with identical results. (**B**) KG-1 cells were treated with 100 nM SNS-032 for 6 h, and then divided into two parts. One was washed into fresh medium without the drug, whereas the other was continuing to culture without washing. The cells were collected at the indicated times for Western blot analysis.

**Figure 4:**
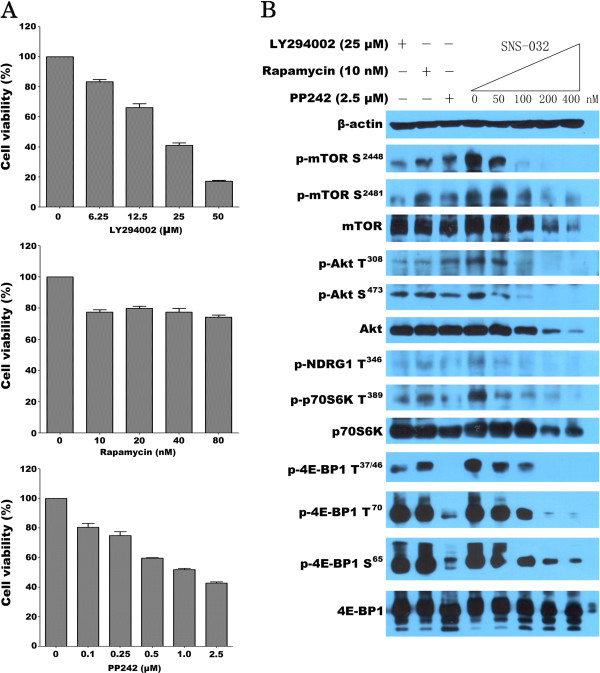
**Effect of SNS-032, PP242, Rapamycin, and LY294002 on phosphorylation of mTORC1 and mTORC2 substrates in HL-60 cells. **(**A**) HL-60 cells were treated with LY294002, Rapamycin, and PP242, respectively, at the indicated doses for 24 h. The anti-proliferative effects were measured by an MTT assay. The values are expressed as means ± SEM from three independent experiments. (**B**) HL-60 cells were treated for 6 h with LY294002 (25 μM), Rapamycine (10 nM), PP242 (2.5 μM), and SNS-032 at 50, 100, 200 and 400 nM, respectively. Whole cell lysates were then subjected to SDS-PAGE followed by immunoblotting with antibody that recognizes the corresponding antigen.

Moreover, SNS-032 also successfully inhibited phosphorylation of 4E-BP1 and p70S6K, the best characterized targets of mTORC1 (Figure 3A). To test the effect of SNS-032 on mTORC2 complex, we examined activity of SGK downstream of mTORC2 by assessing the expression of phosphor-NDRG1 at Thr346. SNS-032 reduced the phosphorylation of NDRG1 in a dose-dependent manner (Figure 2D). Consistently, treatment with this compound significantly decreased the level of phosphor-Akt (Ser473), which is directly downstream of mTORC2, but its inhibitory effect on phosphor-Akt (Thr308) was modest (Figure 3A). To relate the inhibition of activity of mTORC1/mTORC2 with the induction of cell death, we investigated that whether removal of SNS-032 correlates with the recovery from inhibition of phosphor-mTOR and PARP cleavage, a marker of apoptosis (Figure 3B). Immunoblotting analysis revealed that there was a partial restoration of activity of mTORC1 and mTORC2, as well as PRAP cleavage. We next used three kinds of kinase inhibitor LY294002 (targeting PI3K) [15], Rapamycin (targeting mTORC1) [16], and PP242 (targeting mTORC1/mTORC2) [17] as positive controls for the inhibition of mTOR pathway. As shown in Figure 4A, LY294002 (6.25-50 μM) and PP242 (0.1-2.5 μM) inhibited cell growth of HL-60 cells in a dose-dependent fashion. In contrast, Rapamycin (10–80 nM) slightly suppressed cell proliferation. Immunoblotting analysis showed that Rapamycin decreased phosphor-mTOR at Ser2448 and mTORC1 substrates including p70S6K at Thr389 and 4E-BP1 at Thr37/46. Whereas, similar to PP242, SNS-032 significantly inhibited phosphorylation of mTOR at both Ser2448 and Ser2481, and also suppressed phosphorylation of all mTORC1/mTORC2 substrates examined (Figure 4B). Together, these data confirm that SNS-032 not only dephosphorylated Ser2 and Ser5 of RNA polymerase II, it also inhibited phosphorylation of mTOR.

### SNS-032 inhibits IGF-1R and isoform p110δ of PI3K and reduces the mRNA and protein levels of antiapoptotic proteins

**Figure 5:**
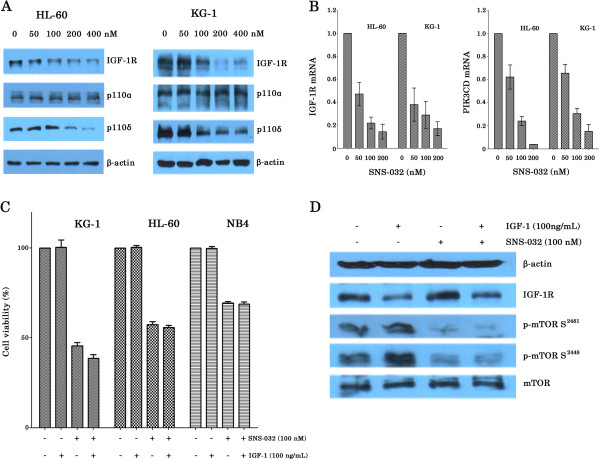
**SNS-032 reduces expression of IGF-1R and PI3K isoform p110δ and IGF-1 does not reverse SNS-032-mediated cellular inhibition. **(**A**) HL-60 and KG-1 cells were cultured with increasing concentrations of SNS-032. At 6 h after treatment, cells were harvested and lysed, and expression of IGF-1R and PI3K isoforms p110α and p110δ was determined by Western blot analysis. Actin was used as protein loading control. (**B**) The levels of IGF-1R and PIK3CD mRNA was determined after 6 hours-exposure of SNS-032 by real-time quantitative RT-PCR, and expressed as relative levels compared with controls. (**C**) Three AML cell lines were treated with 100 nM SNS-032 for 24 h, with or without IGF-1 (100 ng/mL) preincubation for 3 h, and then cell viability was examined by an MTT assay. All values represent the mean of experiments in triplicate; bar, ± s.d. (**D**) HL-60 cells were treated with SNS-032, or combined with IGF-1. Protein expression of IGF-1R, total and phosphorylated forms of mTOR was analyzed by Western blot. Actin levels were used as loading control.

Since there is an autocrine/paracrine stimulation of insulin-like growth factor-1 receptor (IGF-1R) in AML cells, which contribute to activation of PI3K signaling [18], we determined the protein expressions of IGF-1R and class I PI3K isoforms after a 6-hour exposure to increasing concentrations of SNS-032 (Figure 5A). The expression of IGF-1R and p110δ was inhibited by SNS-032 in a dose-dependent fashion. In contrast, p110α protein levels were not changed. The mRNA expression of IGF-1R and p110δ (PIK3CD) was also assessed following treatment with SNS-032 for 6 h using quantitative PCR. IGF-1R and p110δ mRNA expression were significantly inhibited by the drug (Figure 5B), suggesting post-translational effects of SNS-032 on these target proteins. To investigate whether the suppression of IGF-1R and cell death induced by SNS-032 could be causally related, the effects of IGF-1 on SNS-032-induced cell death were examined. As shown in Figure 5C, exposure of cells to 100 ng/mL IGF-1 did not reverse SNS-032-mediated cellular inhibition. In agreement with this result, addition of IGF-1 also did not change inhibition of SNS-032 on phosphorylation of mTOR at both Ser2448 and Ser2481 even though IGF-1 alone upregulated expression of phosphor-mTOR (Figure 5D). These data supported the hypothesis that SNS-032 might directly target mTORC1/mTORC2 pathway.

**Figure 6:**
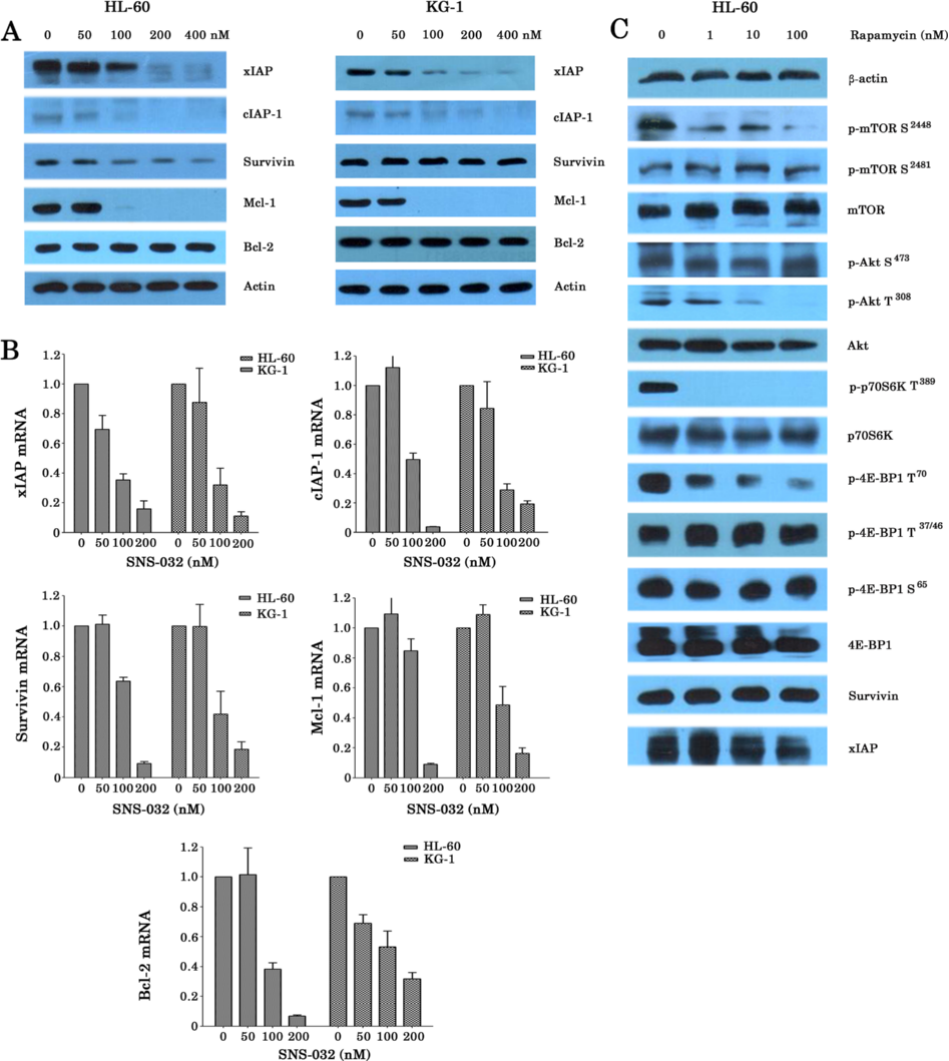
**SNS-032 significantly reduces the expression of anti-apoptotic proteins in AML cells. **(**A**) HL-60 and KG-1 cell lines were treated with SNS-032 at the indicated doses for 6 h. Cell lysates were fractionated on 6-12% SDS-polyacrylamide gels and analyzed by Western blotting with antibodies against xIAP, cIAP-1, Survivin, Mcl-1, Bcl-2 and actin. (**B**) AML cell lines were treated with SNS-032 at the indicated doses. After 6 h treatment, mRNA levels of the indicated genes were measured by real-time RT-PCR. The results are presented as the ratios of mRNA expression in treated cells in relation to the amount present in untreated cells (mean of ± s.d. mRNA from 3 different experiments). (**C**) HL-60 cells were treated with Rapamycin at the indicated doses for 6 h. Equal amounts of protein were resolved by SDS-PAGE and immunoblotted with the indicated antibodies.

The mTORC1 pathway is well known to stimulate protein synthesis [19]. We therefore tested the effects of SNS-032 on the levels of antiapoptotic proteins in HL-60 and KG-1 cell lines using Western blot analyses. Of antiapoptotic proteins, xIAP, cIAP-1, and Mcl-1 were significantly down-regualted and Survivin was slightly inhibited; however, Bcl-2 was unchanged after SNS-032 treatment (Figure 6A). We then measured mRNA expression of these proteins using real time RT-PCR. Consistent with previous reports [8,12], SNS-032 also induced a dose-dependent reduction of mRNA of these genes (range, 84.1-96.3% decrease at 200 nM) for HL-60 cells. Similar results were obtained with KG-1 cells (Figure 6B). We further wished to know whether Rapamycin treatment also reduce anti-apoptotic proteins in AML cells. Western blot analysis showed that this compound slightly downregulated xIAP expression but did not change expression of Survivin. Despite marked reduction of phosphor-mTOR at Ser 2448, Rapamycin upregulated expression of phosphor-Akt (Ser473) (Figure 6C), which might explain why AML cells were relatively resistant to Rapamycin, even at the higher concentration of 80 nM (Figure 4A).

### Perifosine sensitizes AML cell lines and primary cells to SNS-032-mediated cell death

**Figure 7:**
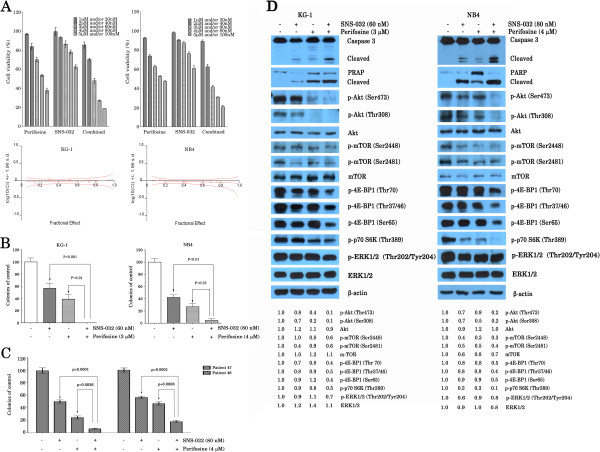
**Combination of SNS-032 with perifosine strongly inhibits phosphor-Akt and enhances cytotoxicity against AML cells. **(**A**) KG-1 and NB4 cells were treated with a series of dilutions of each agent individually and with both compounds simultaneously in a fixed ratio (1:50) for 24 h and cell growth was determined by an MTT assay. Values represent the mean plus or minus s.d. of triplicate experiments. The combination index (CI) was calculated by Calcusyn software (lower panel). (**B**) KG-1 and NB4 cells were treated with SNS-032, perifosine, or combination, and then plated in triplicate, at a density of 2000 cells/ml in methylcellulose medium. Colony counts at 7 days are shown as the average of three independent experiments; bar, ± s.d. (**C**) The primary AML cells from 2 patients were treated with two agents alone or in combination followed by cultured in methylcellulose medium. The number of leukemic colony at 7 days was counted. The data are shown as the means of triplicate experiment. (**D**) AML cell lines were incubated with or without SNS-032, in the presence or absence of perifosine. At 6 h later, cell lysates were prepared, and activation of caspase-3 and cleavage of PARP were examined by Western blot analysis. The expression and phosphorylation of ERK1/2, Akt, mTOR, and its downstream targets were also determined. The difference in the level of protein expression was semi-quantitatively determined by densitometry and expressed as a ratio. Actin was used as protein loading control.

Given the fact that mTOR inhibition activates PI3K/Akt in AML cells [20], we determined whether perifosine, an Akt inhibitor, enhances SNS-032-mediated cell death. For this, we treated KG-1 and NB4 cells with a series of doses of SNS-032 or/and perifosine. As demonstrated in Figure 7A, treatment of KG-1 and NB4 cells with SNS-032 plus perifosine resulted in significantly lower cell viability than either SNS-032 or perifosine treatment. The combination index analysis showed synergistic cytotoxic effects when two drugs were combined at relatively higher concentrations. Next, whether perifosine enhances the effect of SNS-032 in long-term colony formation assay was also examined. We observed that, under the conditions when SNS-032 or perifosine alone had moderate inhibition effect of colony formation of leukemic cell lines the combination therapy almost completely suppressed the colony-forming ability of these leukemic cells (Figure 7B). Similar results were also found in primary blasts obtained from 2 patients with AML (Figure 7C). To further delineate the effect of combination treatment on growth signaling, we examined the effect of SNS-032, perifosine, and combination on the activiation of caspase pathway, phosphorylation of mTOR and downstream targets, as well as expression of phosphor-ERK1/2. As shown in Figure 7D, we found that although SNS-032 and perifosine alone had little effect on caspase 3 and PRAP, the two together were highly effective, suggesting that perifosine can enhance SNS-032-induced apoptosis. Several studies have shown that perifosine inhibits activation of Akt in cancer cells [21,22]. Consistent with these reports, perifosine significantly inhibited the level of phosphorylated Akt (Ser473/Thr308) in KG-1 and NB4 cells and consequently decreased the level of phosphorylated mTOR (Ser2448), which represent the activity of mTORC1, but not that of phosphorylated mTOR (Ser2481). Whereas, phosphorylated mTOR (Ser2481) levels declined in KG-1 and NB4 cells at the low concentrations of 60 and 80 nM of SNS-032, respectively. Importantly, combined SNS-032 and perifosine therapy resulted in almost complete elimination of phosphorylated Akt (Ser473/Thr308) and activity of mTORC1. Consequently, it also significantly attenuated 4E-BP1 phosphorylation at all tested sites (Thr37/46, Thr70, and Ser65) and phosphorylated p70S6K (Thr389), both of which are direct target of mTORC1. Together, this combination treatment is likely to have significant benefit to AML patients as it can synergistically inhibit activity of mTORC1 and Akt in leukemic cells.

## Discussion

CDK inhibitors are gaining success in the clinic as antitumor agents for cancers including hematologic malignancies [11,23]. SNS-032 is a potent CDK inhibitor, which targets CDK2, CDK7, and CDK9, the CDKs that regulate the initiation and elongation of transcription by phosphorylating Ser2 and Ser5 of RNA Pol II, respectively. These biologic effects are attributed to the inhibitory activity against CLL [8,11] and MCL cells [9], which was also demonstrated in AML cells [12]. This study investigated the actions of SNS-032 in AML cells. Our results showed that SNS-032 was active against majority of the tested AML cell lines and primary leukemic cells. However, its mechanisms of action seem to be dependent on the molecular context of the disease. We found that in addition to the typical inhibitory effect on phosphorylation of RNA pol II, SNS-032 caused reduction of activity of mTORC1 and mTORC2, as evidenced by dephosphorylation of mTOR on Ser2448 and Ser2481, without strongly inhibiting PI3K, ERK/MAPK, and STAT3/5. Consistent with these results, SNS-032 treatment elicited potent suppression of phosphorylation 4E-BP1 and p70S6K, the downstream targets of mTORC1, in AML cells and also reduced phosphor-Akt on Ser473, a substrate of mTORC2. Crucially, the effects of SNS-032 in AML cells were partially reversible after drug removal, suggesting the necessity of sustained inhibition of the activity of mTORC1 and mTORC2 for cell killing.

The mTOR is part of two distinct cellular protein complexes, mTORC1 and mTORC2, which plays an important role in the translational control, modulation of metabolic pathways, regulation of cell cycle, and modulation of apoptosis [24]. The constitutive activation of the mTORC1 was found in AML cells, which is independent of PI3K/Akt pathway [25,26]. Also the presence and activity of mTORC2 was demonstrated in the cell lines and primary blasts of AML [27]. Thus, mTORC1/mTORC2 pathways provide a promising target for AML therapy. In fact, the efficacy of rapamycin and its analogs RAD001, CCI-779 (temsirolimus), and AP23573 (deforolimus) that inhibit mTORC1 complex has been investigated in various experimental and clinical studies in AML [28]. Unfortunately, only limited therapeutic effects were observed in clinical trials. The reason for this might be induction of Akt activity because the drugs do not acutely inhibit mTORC2 [20,28,29], and rapamycin is an incomplete inhibitor of mTORC1 [30]. Recently, dual targeting of mTORC1/2 has been demonstrated to be much more effective than treatment with rapamycin in blocking the growth of AML cells and to have potent cytotoxic activity against AML progenitors in vitro [17,27,30,31], suggesting that dual inhibition of mTORC1/2 is a new therapeutic strategy for the treatment of AML. In the present study, the effects on levels of mTOR phosphorylated on Ser2448 and Ser2481 in AML cells by treatment with 200 nM SNS-032 was impressive, with a complete elimination after 6 h of treatment. PI3K signaling pathway is essential for activation of mTOR [28]. Constitutive activation of class I PI3K isoforms has been commonly observed in AML [28]. The expression of p110δ is consistently expressed at a high level in leukemic cells from AML while other isoforms are only up-regulated in the cells from some patients [28,32]. Our studies revealed that 200–400 nM SNS-032 slightly inhibited protein expression of p110δ, but not that of p110α. Moreover, there was decrease in the expression of IGF-1R after exposure to equivalent concentrations of SNS-032. As a constitutively activated IGF-1R is expressed in AML cells and IGF-1/IGF-1R signaling contributes to deregulated PI3K activity [18,33], we investigated whether exogenous IGF-1 stimulation reverses SNS-032-induced cell death. We show here that IGF-1 did not affect not only inhibition of cell growth but also downregulation of phosphor-mTOR at Ser2448 and Ser2481 by SNS-032 in AML cells. Collectively, these data suggest that SNS-032 might directly target mTORC1/mTORC2.

AML is a heterogeneous disease with aberrant regulation of various signal pathways. Thus, simultaneous targeting of two or even more deregulated signal transduction pathways is needed to overcome drug resistance. A recent study of phase I trial of SNS-032 showed that its plasma concentration reached 300 nM when the drug was administered intravenously in the patients with lymphoma who received total doses of 75 mg/m^2^[11]. In this study, we observed that HEL cells were resistant to SNS-032. Meanwhile, Kasumi-1 cells and the primary blasts from a few AML patients were found to be relatively resistant with IC_50_ > 300 nM. The mechanisms by which AML cells are resistance to SNS-032 remain unclear. Given these observations and the fact that mTOR inhibition activates PI3K/Akt in AML cells [20], we postulated that Akt inhibitors might act synergistically with SNS-032 in treating leukemia. Our results show that lower concentrations of perifosine sensitized AML cells to low doses SNS-032-induced cell growth inhibition in vitro. Importantly, perifosine and SNS-032 reduced colony formation ability, which was almost completely eliminated when the two treatments were combined. Moreover, this combination treatment resulted in significant downregulation of phosphor-Akt (Ser 437 and Thr308), compared with using either agent alone. As our results were being prepared for submission, a new report shows that combination of perifosine with mTORC1 inhibitors lead to an enhanced antitumor efficacy in vitro and *in vivo* most likely via activation of GSKβ [34]. Previously, we and other demonstrated that perifosine induced apoptosis in AML cell lines [35] and primary cells [36] but not affect normal CD34^+^ stem cells [36]. Recently, perifosine have entered phase 2 clinical trials for solid tumors and hematologic malignancies including leukemia [28,37]. These data provide a rationale for the combination therapy with SNS-032 and perifosine as a novel approach for treating AML.

## Conclusions

In summary, results in the present study show that SNS-032 is a potential agent for inhibiting cell growth and suppressing of mTORC1/mTORC2 activity in AML cells. Furthermore, synergistic inhibitory effects in vitro by the combination of SNS-032 and Akt inhibitor perifosine were observed at relatively lower concentrations. This combination treatment led to almost complete inhibition of Akt activity. Collectively, we have identified a novel mechanism of action of SNS-032. Our results suggest the possibility of combining SNS-032 with perifosine in a regimen that would optimize the antileukemic activity against cancer cells that are resistant to mTOR inhibitor-induced cell death.

## Materials and methods

### Cell lines, leukemia patient samples, and reagents

Leukemic blasts and normal bone marrow cells were freshly isolated from bone marrow of patients with newly diagnosed, or refractory/relapsed AML (n = 47) and healthy volunteers (n = 5), respectively, after informed consent was obtained using guidelines approved by the Ethics Committee of Zhejiang University the First Affiliated Hospital. CML cell line K562 and AML cell lines HL-60, U937, NB4, THP-1, MV4-11, and HEL were purchased from the American Type Culture Collection (ATCC; Manassas, VA, USA). Kasumi-1 and KG-1 cell lines were gifts from Prof. S Chen (Shanghai Jiaotong University, Shanghai, China) and Prof. R Xu (Zhejiang University, Zhejiang, China), respectively. The primary leukemic cells and cell lines were maintained in Dulbecco modified Eagle medium (DMEM) or RPMI-1640 (Gibco-RRL, Grand Island, NY, USA), respectively, supplemented with heat inactivated fetal bovine serum (FBS) at 37°C in a 5% CO_2_ humidified incubator.

SNS-032 and Rapamycin were purchased from Selleck Chemicals (Houston, TX, USA) and dissolved in dimethylsulfoxide (DMSO) at 1 mg/mL, and then stored at −20°C in small aliquots. Perifosine obtained from Selleck was prepared as a 1 mg/mL stock solution in sterile water and stored at −20°C. IGF-1 was purchased from Peprotech (Rocky Hill, NJ, USA). LY294002 and PP242 were purchased from Sigma (St Louis, MO, USA). Stock solutions of these agents were subsequently diluted with serum-free RPMI-1640 medium prior to use. In all experiments, the final concentration of DMSO did not exceed 0.1%.

### MTT colorimetric survival assay

Cell viability was monitored by 3-(4,5-dimethylthiazol-2-yl) -2,5-diphenyltetrazolium bromide (MTT; Sigma) assay. Briefly, cell lines (2 × 10^4^ cells/well) and primary leukemic cells (1 × 10^5^ cells/well) were seeded in 96-well plates and treated with SNS-032 (50–400 nM) for the indicated times. The end of culture period, 20 μl of MTT solution (5 mg/mL) was added to each well and then the samples were incubated at 37°C for 4 h. The absorbance of the reaction was measured at 570 nm by spectrophotometry. IC_50_ values (the concentration of drug required to kill 50% of the cells) were calculated.

### Colony-forming assay

The effects of SNS-032, perifosine, or combination on the leukemia colony formation (CFU-L) in methylcellulose medium (Sigma) were examined using leukemic colony assay as previously described [38]. Briefly, leukemic cells (2 × 10^3^) in 600 μL of methylcellulose solution were incubated in the presence of the agents or an equivalent amount of medium at 37°C in a humidified atmosphere with 5% CO_2_. Primary leukemic cells were cultured in methylcellulose medium containing recombinant human (rh) stem cell factor (SCF), granulocyte macrophage-colony-stimulating factor (GM-CSF), and interleukin 3 (IL-3, Peprotech) at 2 × 10^4^ cells/dish. After 7 days, CFU-Ls that contain >40 cells were scored manually under a light microscope (Olympus, Tokyo, Japan). For colony assay of human normal bone marrow cells, 3 U/mL rh erythropoietin (Peprotech), 50 ng/mL rhSCF, 30 ng/mL rhGM-CSF, and 10 ng/mL rhIL-3 were added to the methylcellulose medium. The colonies were counted under a microscope on day 12 of culture.

### Flow cytometric analysis

HL-60, KG-1 and HEL cells were treated with SNS-032 at concentrations between 50 and 200 nM for 24 h. Apoptotic cells were quantified by Annexin V-FITC and propidium iodide (PI) double staining using a detection kit purchased from Biouniquer (Jiangsu, China) according to the manufacturer’s instructions.

### Western blot analyses

Cells were incubated for 6 h in the presence or absence of the drugs. The cells were then lysed at 4°C in lysis buffer. Protein concentration was determined by the bicinchoninic acid (BCA) method. The total protein was used for Western blot analysis as previous described [35]. Aliquots containing 50 μg proteins were separated on sodium dodecyl sulfate (SDS)-polyacrylamide gels containing 6-12% acrylamide gradients and then transferred to polyvinylidene difluoride membranes (Millipore, Billerica, USA). The membranes were blocked for 2 h in Tris-buffered saline containing 0.1% Tween and 5% nonfat dry milk and then incubated with primary antibodies overnight at 4°C, followed by incubation with secondary antibodies conjugatesd with fluorescent dyes for 2 h at room temperature. After washing three times, the membranes were incubated with anti-rabbit/mouse IgG conjugated to horseradish peroxidase. The results were visualized with the ECL detecting kit. All primary antibodies were purchased from Cell Signaling Technology (Beverly, MA, USA), except the human anti-RNA poly II, RNA poly II CTD phospho-Ser2 and phospho-Ser5 (Abcam, Cambridge, UK), and phospho-Akt (Thr308), PI3K p110δ (Eptomics, Burlingame, California, USA) primary antibodies.

### Enzyme-linked immunosorbent assay

The enzyme-linked immunosorbent assay (ELISA) to detect endogenous levels of mTOR protein phosphorylated at Ser2448 was performed in 96-well plates using PathScan Phospho-mTOR Sandwich ELISA Kit purchased for Cell Signaling Technology according to the manufacturer’s protocol.

### Real-time PCR

Total RNA was extracted using an RNeasy Plus kit (TaKaRa Shuzo, Kyoto, Japan). Each cDNA template was made from total RNA with reverse transcriptase kit according to manufacturer’s instructions (Invitrogen, Carlsbad, CA, USA). Amplification reactions were performed using SYBR® Premix Ex Taq™ (TaKaRa Shuzo) in a 25 μL volume on a 96-well optical reaction plate in the iQ5 Multicolor Real-time PCR Detection System (Bio-Rad, Hercules, CA, USA). The following cycling parameters were used: 30 seconds at 95°C for initial denaturing, 5 seconds at 95°C for denaturing and 30 seconds at 60°C for annealing and extension for the total of 40 cycles. The fold change in mRNA was calculated by the 2^-ΔΔCt^ method. All samples were normalized to 18 s ribosomal RNA, an RNA polymerase I transcript that is not modulated by inhibition of RNA pol II. Primer sequences were shown in Table 2.

**Table 2:** Sense and antisense primers for amplification of the tested genes with real-time PCR

**mRNA target**	**Sense (5**^**′**^**) primers**	**Antisense (3**^**′**^**) primers**
xIAP	5^′^-CCATATACCCGAGGAACCCT-3^′^	5^′^-TTTCCACCACAACAAAAGCA-3^′^
Mcl-1	5^′^-AAAAGCAAGTGGCAAGAGGA-3^′^	5^′^-TTAATGAATTCGGCGGGTAA-3^′^
Bcl-2	5^′^-AAGATTGATGGGATCGTTGC-3^′^	5^′^-TGTGCTTTGCATTCTTGGAC-3^′^
cIAP-1	5^′^-GCTCAGTAACTGGGAACCAAA-3^′^	5^′^-ATCATTGCGACCCACATAATA-3^′^
Survivin	5^′^- CAGATTTGAATCGCGGGACCC-3^′^	5^′^- CCAAGTCTGGCTCGTTCTCAG-3^′^
IGF-1R	5^′^-TTAAAATGGCCAGAACCTGAG-3^′^	5^′^-ATTATAACCAAGCCTCCCAC-3^′^
PIK3CD	5^′^-CGGGACACAGGGAAGTTCAGGT-3^′^	5^′^-TAAGGAGTCAGGCCAGGGCGG-3^′^
18 s rRNA	5^′^-GTAACCCGTTGAACCCCATT-3^′^	5^′^-CCATCCAATCGGTAGTAGCG-3^′^

### Statistical analysis

One-way analysis of variance followed by the Tukey test, or Student’s test was performed using the GraphPad Prism 5.0. *P*-values that were less than 0.05 were considered statistically significant. Synergisms in the combination treatments were analyzed using CalcuSyn software (Biosoft, Cambridge, UK). The data were expressed as log_10_ (CI) versus fraction affected. By this method, log_10_ (CI) <0 indicates a synergistic.

## Competing interests

The authors declare that they have no competing interests.

## Authors’ contributions

WBQ designed and guided the experiments, HTM, YMJ, HL, LSY, CMY, and XY conducted the experiments, WBQ and YMJ analyzed the results and wrote the manuscript. All authors read and approved the final manuscript.

## Authors’ information

Haitao Meng and Yingming Jin are co-first author.

## References

[B1] Burnett AK Hills RK Milligan DW Goldstone AH Prentice AG McMullin MF Duncombe A Gibson B Wheatley K Attempts to optimize induction and consolidation treatment in acute myeloid leukemia: results of the MRC AML12 trial J Clin Oncol 2010 28 586 595 10.1200/JCO.2009.22.9088 20038732

[B2] Büchner T Berdel WE Haferlach C Haferlach T Schnittger S Müller-Tidow C Braess J Spiekermann K Kienast J Staib P Age-related risk profile and chemotherapy dose response in acute myeloid leukemia: a study by the German acute myeloid leukemia cooperative group J Clin Oncol 2009 27 61 69 19047294 10.1200/JCO.2007.15.4245

[B3] Scholl C Gilliland DG Fröhling S Deregulation of signaling pathways in acute myeloid leukemia Semin Oncol 2008 35 336 345 10.1053/j.seminoncol.2008.04.004 18692684

[B4] Zhu X Ma Y Liu D Novel agents and regimens for acute myeloid leukemia: 2009 ASH annual meeting highlights J Hematol Oncol 2010 23 3 17 10.1186/1756-8722-3-17 PMC2880983 20416083

[B5] Roboz GJ Novel approaches to the treatment of acute myeloid leukemia Hematology Am Soc Hematol Educ Program 2011 2011 43 50 10.1182/asheducation-2011.1.43 22160011

[B6] Senderowicz AM Inhibitors of cyclin-dependent kinase modulators for cancer therapy Prog Drug Res 2005 63 183 206 10.1007/3-7643-7414-4_8 16265881

[B7] Dickson MA Schwartz GK Development of cell-cycle inhibitors for cancer therapy Curr Oncol 2009 16 36 43 19370178 10.3747/co.v16i2.428 PMC2669234

[B8] Chen R Wierda WG Chubb S Hawtin RE Fox JA Keating MJ Gandhi V Plunkett W Mechanism of action of SNS-032, a novel cyclin-dependent kinase inhibitor, in chronic lymphocytic leukemia Blood 2009 113 4637 4645 10.1182/blood-2008-12-190256 19234140 PMC2680368

[B9] Chen R Chubb S Cheng T Hawtin RE Gandhi V Plunkett W Responses in mantle cell lymphoma cells to SNS-032 depend on the biological context of each cell line Cancer Res 2010 70 6587 6597 10.1158/0008-5472.CAN-09-3578 20663900 PMC2929954

[B10] Wu Y Chen C Sun X Shi X Jin B Ding K Yeung SC Pan J Cyclin-dependent kinase 7/9 inhibitor SNS-032 abrogates FIP1-like-1 platelet-derived growth factor receptor α and bcr-abl oncogene addiction in malignant hematologic cells Clin Cancer Res 2012 18 1966 1978 10.1158/1078-0432.CCR-11-1971 22447844

[B11] Tong WG Chen R Plunkett W Siegel D Sinha R Harvey RD Badros AZ Popplewell L Coutre S Fox JA Phase I and pharmacologic study of SNS-032, a potent and selective Cdk2, 7, and 9 inhibitor, in patients with advanced chronic lymphocytic leukemia and multiple myeloma J Clin Oncol 2010 28 3015 3022 10.1200/JCO.2009.26.1347 20479412 PMC4979218

[B12] Walsby E Lazenby M Pepper C Burnett AK The cyclin-dependent kinase inhibitor SNS-032 has single agent activity in AML cells and is highly synergistic with cytarabine Leukemia 2011 25 411 419 10.1038/leu.2010.290 21212792

[B13] Park S Chapuis N Tamburini J Bardet V Cornillet-Lefebvre P Willems L Green A Mayeux P Lacombe C Bouscary D Role of the PI3K/AKT and mTOR signaling pathways in acute myeloid leukemia Haematologica 2010 95 819 828 10.3324/haematol.2009.013797 19951971 PMC2864389

[B14] Oh WJ Jacinto E mTOR complex 2 signaling and functions Cell Cycle 2011 10 2305 2316 10.4161/cc.10.14.16586 21670596 PMC3322468

[B15] Chapuis N Tamburini J Green AS Vignon C Bardet V Neyret A Pannetier M Willems L Park S Macone A Dual inhibition of PI3K and mTORC1/2 signaling by NVP-BEZ235 as a New therapeutic strategy for acute myeloid leukemia Clin Cancer Res 2010 16 5424 5435 10.1158/1078-0432.CCR-10-1102 20884625

[B16] Gupta M Hendrickson AE Yun SS Han JJ Schneider PA Koh BD Stenson MJ Wellik LE Shing JC Peterson KL Dual mTORC1/mTORC2 inhibition diminishes Akt activation and induces Puma-dependent apoptosis in lymphoid malignancies Blood 2012 119 476 487 10.1182/blood-2011-04-346601 22080480 PMC3257013

[B17] Zeng Z Shi YX Tsao T Qiu Y Kornblau SM Baggerly KA Liu W Jessen K Liu Y Kantarjian H Targeting of mTORC1/2 by the mTOR kinase inhibitor PP242 induces apoptosis in AML cells under conditions mimicking the bone marrow microenvironment Blood 2012 120 2679 2689 10.1182/blood-2011-11-393934 22826565 PMC3460689

[B18] Wahner Hendrickson AE Haluska P Schneider PA Loegering DA Peterson KL Attar R Smith BD Erlichman C Gottardis M Karp JE Expression of insulin receptor isoform A and insulin-like growth factor-1 receptor in human acute myelogenous leukemia: effect of the dual-receptor inhibitor BMS-536924 in vitro Cancer Res 2009 69 7635 7643 10.1158/0008-5472.CAN-09-0511 19789352 PMC2762752

[B19] Dennis MD Jefferson LS Kimball SR Role of p70S6K1-mediated phosphorylation of eIF4B and PDCD4 in the regulation of protein synthesis J Biol Chem 2012 287 42890 42899 10.1074/jbc.M112.404822 23105104 PMC3522285

[B20] Tamburini J Chapuis N Bardet V Park S Sujobert P Willems L Ifrah N Dreyfus F Mayeux P Lacombe C Mammalian target of rapamycin (mTOR) inhibition activates phosphatidylinositol 3-kinase/Akt by up-regulating insulin-like growth factor-1 receptor signaling in acute myeloid leukemia: rationale for therapeutic inhibition of both pathways Blood 2008 111 379 382 10.1182/blood-2007-03-080796 17878402

[B21] Pinton G Manente AG Angeli G Mutti L Moro L Perifosine as a potential novel anti-cancer agent inhibits EGFR/MET-AKT axis in malignant pleural mesothelioma PLoS One 2012 7 e36856 10.1371/journal.pone.0036856 22590625 PMC3349630

[B22] Li Z Tan F Liewehr DJ Steinberg SM Thiele CJ In vitro and *In vivo* inhibition of Neuroblastoma tumor cell growth by AKT inhibitor Perifosine J Natl Cancer Inst 2010 102 758 770 10.1093/jnci/djq125 20463309 PMC2879416

[B23] Bose P Perkins EB Honeycut C Wellons MD Stefan T Jacobberger JW Kontopodis E Beumer JH Egorin MJ Imamura CK Phase I trial of the combination of flavopiridol and imatinib mesylate in patients with Bcr-Abl + hematological malignancies Cancer Chemother Pharmacol 2012 69 1657 1667 10.1007/s00280-012-1839-5 22349810 PMC3365614

[B24] Vignot S Faivre S Aguirre D Raymond E mTOR-targeted therapy of cancer with rapamycin derivatives Ann Oncol 2005 16 525 537 10.1093/annonc/mdi113 15728109

[B25] Tamburini J Green AS Bardet V Chapuis N Park S Willems L Uzunov M Ifrah N Dreyfus F Lacombe C Protein synthesis is resistant to rapamycin and constitutes a promising therapeutic target in acute myeloid leukemia Blood 2009 114 1618 1627 10.1182/blood-2008-10-184515 19458359

[B26] Xu Q Thompson JE Carroll M mTOR regulates cell survival after etoposide treatment in primary AML cells Blood 2005 106 4261 4268 10.1182/blood-2004-11-4468 16150937 PMC1895255

[B27] Altman JK Sassano A Kaur S Glaser H Kroczynska B Redig AJ Russo S Barr S Platanias LC Dual mTORC2/mTORC1 targeting results in potent suppressive effects on acute myeloid leukemia (AML) progenitors Clin Cancer Res 2011 17 4378 4388 10.1158/1078-0432.CCR-10-2285 21415215 PMC3131493

[B28] Polak R Buitenhuis M The PI3K/PKB signaling module as key regulator of hematopoiesis: implications for therapeutic strategies in leukemia Blood 2012 119 911 923 10.1182/blood-2011-07-366203 22065598

[B29] Guertin DA Sabatini DM The pharmacology of mTOR inhibition Sci Signal 2009 2 67 pe24 Apr 21 10.1126/scisignal.267pe24 19383975

[B30] Janes MR Limon JJ So L Chen J Lim RJ Chavez MA Vu C Lilly MB Mallya S Ong ST Effective and selective targeting of leukemia cells using a TORC1/2 kinase inhibitor Nat Med 2010 16 205 213 10.1038/nm.2091 20072130 PMC4017764

[B31] Willems L Chapuis N Puissant A Maciel TT Green AS Jacque N Vignon C Park S Guichard S Herault O The dual mTORC1 and mTORC2 inhibitor AZD8055 has anti-tumor activity in acute myeloid leukemia Leukemia 2012 26 1195 1202 10.1038/leu.2011.339 22143671

[B32] Sujobert P Bardet V Cornillet-Lefebvre P Hayflick JS Prie N Verdier F Vanhaesebroeck B Muller O Pesce F Ifrah N Essential role for the p110delta isoform in phosphoinositide 3-kinase activation and cell proliferation in acute myeloid leukemia Blood 2005 106 1063 1066 10.1182/blood-2004-08-3225 15840695

[B33] Chapuis N Tamburini J Cornillet-Lefebvre P Gillot L Bardet V Willems L Park S Green AS Ifrah N Dreyfus F Autocrine IGF-1/IGF-1R signaling is responsible for constitutive PI3K/Akt activation in acute myeloid leukemia: therapeutic value of neutralizing anti-IGF-1R antibody Haematologica 2010 95 415 423 10.3324/haematol.2009.010785 20007139 PMC2833071

[B34] Ma Z Zhu L Luo X Zhai S Li P Wang X Perifosine enhances mTORC1-targeted cancer therapy by activation of GSK3β in NSCLC cells Cancer Biol Ther 2012 13 1009 1017 10.4161/cbt.20989 22825337 PMC3461807

[B35] Tong Y Liu YY You LS Qian WB Perifosine induces protective autophagy and upregulation of ATG5 in human chronic myelogenous leukemia cells in vitro Acta Pharmacol Sin 2012 33 542 550 10.1038/aps.2011.192 22407228 PMC4003362

[B36] Papa V Tazzari PL Chiarini F Cappellini A Ricci F Billi AM Evangelisti C Ottaviani E Martinelli G Testoni N Proapoptotic activity and chemosensitizing effect of the novel Akt inhibitor perifosine in acute myelogenous leukemia cells Leukemia 2008 22 147 160 10.1038/sj.leu.2404980 17928881

[B37] Pal SK Reckamp K Yu H Figlin RA Akt inhibitors in clinical development for the treatment of cancer Expert Opin Investig Drugs 2010 19 1355 1366 10.1517/13543784.2010.520701 PMC3244346 20846000

[B38] Jin J Liu H Yang C Li G Liu X Qian Q Qian W Effective gene-viral therapy of leukemia by a new fiber chimeric oncolytic adenovirus expressing TRAIL: in vitro and *in vivo* evaluation Mol Cancer Ther 2009 8 1387 1397 10.1158/1535-7163.MCT-08-0962 19417152

